# Systematic reconstruction of binding and stability landscapes of the fluorogenic aptamer spinach

**DOI:** 10.1093/nar/gkv944

**Published:** 2015-09-22

**Authors:** Simon Ketterer, David Fuchs, Wilfried Weber, Matthias Meier

**Affiliations:** 1 Microfluidic and Biological Engineering, Department of Microsystems Engineering - IMTEK, University of Freiburg, Georges-Koehler-Allee 103, 79110 Freiburg, Germany; 2 Centre for Biological Signalling Studies - BIOSS, University of Freiburg, Schänzlestrasse 18, 79104 Freiburg, Germany; 3 Faculty of Biology, University of Freiburg, Schänzlestrasse 18, 79104 Freiburg, Germany

## Abstract

Fluorogenic RNAs that are based on the complex formed by 3,5-difluoro-4-hydroxybenzylidene imidazolinone (DFHBI) derivatives and the RNA aptamer named Spinach were used to engineer a new generation of *in vitro* and *in vivo* sensors for bioanalytics. With the resolved crystal structure of the RNA/small molecule complex, the engineering map becomes available, but comprehensive information regarding the thermodynamic profile of the molecule is missing. Here, we reconstructed the full thermodynamic binding and stability landscapes between DFHBI and a truncated sequence of first-generation Spinach. For this purpose, we established a systematic screening procedure for single- and double-point mutations on a microfluidic large-scale integrated chip platform for 87-nt long RNAs. The thermodynamic profile with single base resolution was used to engineer an improved fluorogenic spinach generation via a directed rather than evolutional approach.

## INTRODUCTION

Genetically encoded fluorescent RNAs have opened up new possibilities for *in situ* imaging of RNAs (1–3). Fluorescent RNA modules are comprised of an aptamer and a small fluorogenic chemical, and fluorescence is observed only upon binding of the aptamer to the fluorogen. The aptamer ‘Spinach’ was engineered with specific affinity for the fluorogenic compound 3,5-difluoro-4-hydroxybenzylidene imidazolinone (DFHBI), which is membrane permeable and nontoxic to cells (4). The structure of the DFHBI and Spinach complex resembles the inner core of green fluorescent protein (GFP); thus, it emits green fluorescence with a quantum yield comparable to that of GFP (1,5). Since the discovery of the Spinach successors, fluorogenic aptamers with similar sequences have been developed to overcome the initial problems related to temperature and *in situ* stability or to improve optical properties of the Spinach-ligand complex (4,6–8). One of the most prevailing uses of Spinach is the possibility to construct conditionally fluorescent sensors for small molecules by merging the Spinach RNA sequence to other aptamers (9–14).

Effective engineering of the RNA-small-molecule interactions requires the information about the molecular structure and the thermodynamics of the binding and stability landscapes of Spinach with its fluorogenic compound. The structures of short RNAs are predictable on the basis of their sequences, but for longer RNA strands, structural prediction algorithms often fail. This problem was evident for Spinach, where the crystal structure (5,15) showed strong deviations from the predicted two-dimensional (2D) structure. Folding and stability of RNA structures are predominantly governed by the free energies of the secondary structure elements. The interplay between these motifs and their thermodynamic contribution to the small molecule binding reaction has to be determined for each aptamer. The first Spinach generation exhibits three stems, a tetra-loop and a guanine-quadruplex structure element. Structural analysis revealed that the binding motif for DFHBI within Spinach is formed between a RNA duplex and quadruplex junction. The thermodynamic contribution of the secondary structure elements to the binding and stability of Spinach has not been fully revealed.

It is clear that knowledge of the binding and stability thermodynamics of fluorogenic RNAs is critical for the engineering process of these molecules. Enthalpy and entropy contributions of DFHBI binding to Spinach are of general biophysical interest since they define the photochemical properties of Spinach. A wide range of methods for scanning mutagenesis are currently being developed to study the sequence dependence of RNA-protein interactions (16,17). The current focus is on next-generation sequencing (NGS) technology because of its high throughput. Previously, we developed a microfluidic large-scale integration chip (mLSI) integrating the chemical workflow for RNA scanning mutagenesis. The advantage of microfluidic technology in comparison to NGS is its flexibility in terms of adjustments to chemical-physical parameters within a wide range of molecular binding conditions (18). Previously, the chip was exploited to establish the complete binding landscape between a short RNA stem-loop (16 nt) and a cognate stem-loop binding protein (19). Here, we demonstrate that mLSI technology can be used to systematically study the interactions between RNA aptamers and small molecules. For this purpose, 228 single-point mutations (SPMs), 134 double-point mutations (DPMs), of a Spinach variant were *in vitro* synthesized in 640 1 nl sized reaction compartments on chip. With the miniaturized pull-down assay, control over the chip temperature, and DFHBI concentrations, we were able to record the full thermodynamic and stability profile of Spinach. For this, the thermodynamic profiles of the Spinach mutants were mapped onto the crystal structure. Further, with the complete thermodynamic profile, we developed an improved version of the Spinach aptamer. Our general systematic approach of pinpointing the contribution of single nucleotides to a fluorogenic aptamer stability, function, free energy and enthalpy of binding presents an efficient method to rapidly refine successor molecules for analytical applications.

## MATERIALS AND METHODS

### Spinach DNA template library construction

All DNA Spinach mutants were constructed from two DNA oligonucleotides O1 and O2 (Sigma Aldrich, Germany), which share an overlapping region of 19 nt (see Supplementary Figure S1). Additionally, O1 carried a T7 promoter, a DNA pull-down anchor and probe sequence (see Supplementary Table S1). The two oligonucleotides were combined by PCR (20), in which 20 nM of O1 and O2 were first annealed and complemented with a Hot Start DNA polymerase (New England Biolabs, USA). Subsequently, a forward and reverse primer (200 nM) was added and the construct amplified by PCR. Upon combination of the differently mutated O1 and O2 oligonucleotides, the SPMs and DPMs of Spinach were generated. For a negative control the O1 alone was taken, which missed the Spinach core sequence. Purified PCR samples were dissolved in 1x SSC buffer with 1% BSA at a DNA concentration of ∼100 ng/ml. The DNA of the Spinach mutants were then spotted onto an epoxy coated microscope slide (Cel-1, USA) with a microarray contact printer (OmniGrid).

### Microfluidic chip

Polydimethylsiloxane (PDMS) chips were manufactured following the standard procedure for multilayer devices (21). In short, flow and control molds were fabricated by using SU-8 3025 (MicroChem, USA) and AZ 9260 (MicroChemicals, Germany), respectively. The feature height of the control mold was 25 ± 1 μm and for the flow mold 18 ± 1 μm. PDMS (Sylgard 184, from Dow Corning, USA) was cast onto both molds and hardened for 20 min at 80°C. Bonding between the flow and control PDMS layer was achieved by using off-ratio method. Assembled PDMS devices were aligned to the spotted epoxy-coated glass slide and thermally bonded on a hot plate for 6 h at 80°C. Automated perfusion operations on chip included the following sequential steps: (i) coating of the epoxy-coated glass slide with biotinylated BSA (Thermo Scientific, Germany), (ii) NeutrAvidin (Thermo Scientific, Germany), (iii) ROX-labeled anchor DNA with conjugated biotin at a concentration of 10 μM (Sigma Aldrich, Germany). Local deposition of the Spinach pull-down anchor within each unit cell of the microfluidic chip was achieved with help of a pneumatic membrane valve (18,22). The valve protected a circular surface area with a diameter of ∼60 μm during a passivation step of the reactive NeutrAvidin. The transcription of Spinach DNA mutants was initiated upon introduction of an *in vitro* transcription (IVT) solution (HiScribe T7 High Yield RNA Synthesis Kit, New England Biolabs, USA) into the 640 microchambers containing the DNA spots. During a 2 h incubation step the chip surface was heated to 37°C using an ITO heating glass slide (Tokai Hit, Japan). Transcribed Spinach mutant RNAs were allowed to diffuse to the pull-down area in the separated unit cells and non-hybridized RNAs were washed from the unit cells.

### Determination of the spinach dissociation constants on chip

In order to obtain the dissociation constants between DFHBI and the Spinach mutants, 12 different concentrations of DFHBI (0.435–43.5 μM) dissolved in detection buffer (40 mM HEPES, 125 mM KCl, 5 mM MgCl2, 5% DMSO final) were sequentially introduced into the chip. At each concentration step the DFHBI was equilibrated for 10 min. The chip was then imaged at excitation wavelength of 470 nm (Spinach signal) and 555 nm (pull-down anchor signal) on a Zeiss–Axio Observer microscope. After the last titration step the total amount of the Spinach mutants on the pull-down areas were determined with a fluorescence detection probe. The Cy-5-labeled probe (Excitation 625 nm) was dissolved at a concentration of 10 μM in 1x PBS. The binding enthalpy of DFHBI to the Spinach mutants were obtained upon repeating the concentration experiment at three different temperatures, i.e. 19°C, 23°C and 28°C.

### Determination of apparent melting temperatures of the spinach/DFHBI complex on chip

In order to obtain the apparent/of the RNA-DFHBI complex, $T_m^{ap}$ values of the Spinach mutants, a DFHBI concentration of 43.5 μM in detection buffer was introduced into the microfluidic chip after IVT. In the following the chip temperature was increased from 23°C to 49°C in 2°C steps. At each temperature step the chip was imaged. An intermediate incubation step of 10 min was sufficient to obtain a stable fluorescence signal. Image acquisition was performed in accordance to the dissociation experiments.

### Determination of DFHBI concentration

The DFHBI concentration (Lucerna, NY) was calculated by measuring the absorbance with Unicam UV-300 (molar extinction coefficient of DFHBI 30 100 M^−1^ cm^−1^) (6).

### Image analysis

The fluorescence images were evaluated with Matlab (Mathworks, USA). First, the pull-down areas were segmented using the oligonucleotide anchor signal (Ex. 555 nm). A generalized 2D Gaussian function was fitted to the fluorescence image to define the pixel of the circular pull-down area (foreground). For correction of the local background the pixel positions of a disk surrounding the pull-down area were determined. The radius of this disk was 1.5 times the radius of the circular pull-down area. The foreground and background pixel positions were then used to extract the median of the Spinach/DFHBI-fluorescence signal, *S_i_*, the fluorescence background, *S*_b_, and the Cy5-probe signal, *S_p_*. Spinach/DFHBI signal of all mutants, *S_m_*, were background corrected and normalized to the total amount of RNA on the pull-down area as $S_m = (S_i - S_b )/S_p$.

### Binding model

The thermodynamic binding of Spinach, *[S]*, and DFHBI, *[D]*, to the Spinach/DFHBI complex, *[SD]*, is described by the two-state binding model with the dissociation constant *K_d_*:

(1) \begin{equation*} S + D \mathop{\rightleftharpoons} SD;K_d = \frac{{\left[ S \right] \cdot \left[ D \right]}}{{\left[ {SD} \right]}} \end{equation*}

The fluorescence signal obtained from the chip pull-down areas corresponds to the concentration of the Spinach/DFHBI complex [*SD*]:

(2) \begin{equation*} \frac{{S_m }}{{S_m^{max} }} = \frac{{\left[ {SD} \right]}}{{\left[ S \right] + \left[ {SD} \right]}} = \frac{{\left[ D \right]/K_d }}{{1 + \left[ D \right]/K_d }}\end{equation*}

where the fluorescence signals of the Spinach mutant/DFHBI complexes within the titration experiments were normalized to the fluorescence signal of the Spinach mutant/DFHBI complexes at the highest DFHBI concentration, $S_m^{max}$. The effect of the mutation on wild type Spinach is given by the difference of the free binding energy of the mutant, Δ*G_m_*, and wild type Spinach, Δ*G_wt_*, to DFHBI,

(3) \begin{eqnarray*} &&{\rm \Delta \Delta }G\left( T \right) = \nonumber \\ && {\rm \Delta }G_m \left( T \right) - {\rm \Delta G}_{wt} \left( T \right) = - RT{\rm ln}\left( {K_d^m /K_d^{wt} } \right)\end{eqnarray*}

where *R, T*, $K_d^m$ and $K_d^{wt}$, denote for the molar gas constant, temperature, dissociation constants of the mutant, and wild type Spinach to DFHBI, respectively. The binding enthalpy, Δ*H*, is given by the Van't Hoff equation,

(4) \begin{equation*} \ln \left( {K_d } \right) = \frac{{ - {\rm \Delta }H}}{{RT}} + \frac{{{\rm \Delta }S}}{R}\end{equation*}

where Δ*S* denote binding entropy. The binding enthalpy for each Spinach mutant to DFHBI is obtained from a $\ln \left( {K_d } \right)$ versus $1/T$ plot with linear regression analysis. Likewise, the effect of base mutations in Spinach on the binding enthalpy, Δ*H_m_*, to DFHBI is compared to the wild type Spinach binding enthalpy, Δ*H_wt_*, by ${\rm \Delta \Delta }H = {\rm \Delta }H_m - {\rm \Delta H}_{wt}$.

### Folding model

The apparent melting temperature of the RNA/DFHBI complex, $T_m^{ap}$, is derived from the free energy change ${\rm \Delta }G_T = {\rm \Delta }H_T - T{\rm \Delta }S_T$ of a two-state reaction between the folded, *F*, and unfolded, *U*, Spinach-DFHBI complex. The equilibrium constant of this reaction is given by

(5) \begin{equation*} k_T = \frac{{\left[ F \right]}}{{\left[ U \right]}} = e^{ - \frac{{{\rm \Delta }G_T }}{{RT}}} \end{equation*}

Based on this equilibrium constant, the fluorescence signal distribution depending on the temperature is expressed as

(6) \begin{equation*} \frac{{S_m }}{{S_{m,T}^{max} }} = \frac{{\left[ F \right]}}{{\left[ U \right] + \left[ F \right]}} = \frac{1}{{1 + e^{\frac{{{\rm \Delta }H_T }}{R} \cdot \left( {\frac{1}{{{\rm }T_m^{ap} }} - \frac{1}{T}} \right)} }}\end{equation*}

where $T_m^{ap}$ is ${\rm \Delta }H_T /{\rm \Delta }S_T$. The fluorescence signals of the Spinach mutant/DFHBI complexes within the unfolding experiments were normalized to the fluorescence signal of Spinach mutant/DFHBI complexes at the lowest temperature, $S_m^{low}$.

### Scoring model

To obtain the local contribution of the SPM *n* to the thermodynamic and structural landscape, the four thermodynamic parameters (${\rm \Delta }G$.,${\rm S}_m$,${\rm \Delta }H$., $T_m$) were normalized to $X_i \left( {i = 1.4} \right)$ by

(7) \begin{equation*} X_i = \frac{{(x_i - x_{min} )}}{{(x_{max} - x_{min} )}}\end{equation*}

where $x_i \left( {i = 1.4} \right)$ denote for any of the four thermodynamic parameters. $x_{min}$ and $x_{max}$ are the minimum and maximum value found among all SPMs for the corresponding thermodynamic parameter, respectively (Supplementary Table S3). The $X_i$ score for the SPM, which lost their fluorogenic property, were set to −1. The four single scores were then combined by

(8) \begin{equation*} S_j = \mathop \sum \nolimits_{i = 1}^4 X_i \end{equation*}

to an overall thermonamic score, $S_j$ for each SPM. Finally, a moving average function over the nearest SPM neighbors in 5′ and 3′ direction was applied:

(9) \begin{equation*} Score\left( n \right) = \frac{1}{3}\mathop \sum \nolimits_{j = n - 1}^{n + 1} S_j \end{equation*}

### Error calculation

Errors, $\sigma$, for the Snach fluorescence signals, *S*_m_, are given as standard errors. For this four repeats were measured for each mutant. Errors for ${\rm \Delta }G$, ${\rm \Delta }H$, and$T_m^{ap}$ values were calculated according to:

(10) \begin{equation*} \sigma = \sqrt {\left( {\frac{{\sum\nolimits_{j = 1}^N {\left( {X_j - \bar X} \right)^2 } }}{{(N - 1) \cdot N}}} \right)^2 + \left( {\frac{1}{N}\sum\nolimits_{j = 1}^N {\sigma _j^2 } } \right)^2 } \end{equation*}

where the first term contains the error of the repeats and the second term the error of fitting the data to the corresponding models described above. Thereby, $\bar X$ is the mean value of the fit results, and $X_j$ is the value of fit results, and $\sigma _j$ is the fit error.

## RESULTS AND DISCUSSION

### Structural analysis

**Figure 1.**
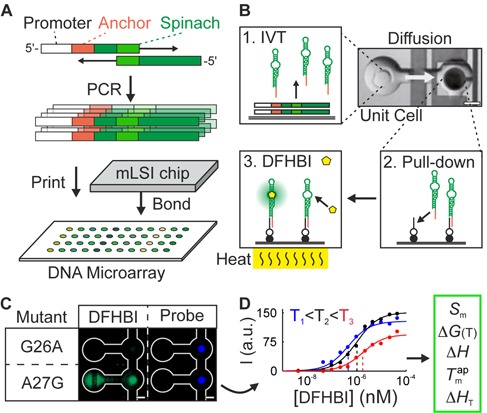
The experimental workflow to determine binding and stability landscapes of fluorescent RNA aptamers. (**A**) A DNA mutant library was created by stitch PCR. DNA sequences encoding of the single- and double-point Spinach mutants were spotted on a glass microarray and bonded to a microfluidic polydimethylsiloxane (PDMS) chip. (**B**) On the chip, the DNA transcription (1), a miniaturized pull-down assay (2), and a perfusion system (3) for 640 RNA mutants were automated in separate microfluidic unit cells. The chip was mounted on a thin glass coated with indium tin oxide (ITO) for control of the temperature on the glass surface. (**C**) Fluorescent signals of two Spinach mutants in the pull-down area of a chip unit cell. The total amount of RNA in the pull-down area was quantified with a probe molecule. (**D**) The binding and melting curves were used to calculate the free energy (Δ*G*), and enthalpy (Δ*H*) of the binding, the apparent melting temperature of the RNA–DFHBI complex ($T_m^{ap}$), and the fluorescence intensity of the Spinach complexes (*S*) with 3,5-difluoro-4-hydroxybenzylidene imidazolinone (DFHBI).

The analytical workflow for determining the binding and stability landscape is depicted in Figure 1. The procedure starts with the synthesis of all SPMs and selected DPMs at the DNA level by PCR (20). As wild type (*wt*) Spinach, we used a sequence based on the previously published 98 nt 24–2 Spinach sequence. Within the sequence, we introduced the SPM A6G. The beneficial property of this mutation was previously reported in the variant Spinach 1.2 since it corrects for a Watson–Crick base pair mismatch (4). Further, in difference to the original Spinach 24–2 sequence the nucleotide 37–43 and 56–59 we deleted. The reason for this was to shorten the length of the Spinach sequence for *in vitro* synthesis based on the initial Spinach publication, these nucleotides were not important for the fluorescence properties of Spinach (1). The resulting 87-nucleotide *wt* Spinach sequence was divided into two fragments, where the 5′ and the 3′ fragment overlapped by 19 nt (Supplementary Figure S1). Spinach molecules with mutations in the 5′ or 3′ fragment outside the overlapping sequence were assembled and amplified using standard PCR and the corresponding wild-type (WT) fragment. For assembly of Spinach molecules with point mutations in the overlap region, we used the 5′ and 3′ fragments both carrying the mutations. The 5′ fragments contained the T7 promoter for IVT and a capture sequence for pulling down the Spinach RNA after transcription. Combinatorial synthesis of the DNA library reduced the cost and error rate associated with long oligonucleotides. The resulting DNA mutation library of Spinach was spotted onto an epoxy-coated glass slide and bonded to a previously designed platform for mLSI chips for testing bimolecular interactions.

In brief, the mLSI chip features 640 functional unit cells for IVT of RNA molecules together with a miniaturized pull-down assay (Figure 1B). Fluidic channel networks and control logic on multilayer PDMS chip allowed us to analyze the unit cells sequentially by means of fluids. First, in each unit cell, a pull-down assay for the RNA aptamer was constructed on the epoxy glass surface by specifically depositing NeutrAvidin within a circular region with a diameter of 100 μm. This task was accomplished with the help of pneumatic membrane valves. A biotin-labeled oligonucleotide with a sequence reverse-complementary to the 5′ capture tag on all Spinach mutants was then bound to the NeutrAvidin in all unit cells. In the following the spotted DNA was re-suspended with IVT solution. Upon raising the chip temperature to 37°C, the Spinach RNA mutants were generated within the separated unit cells. Free diffusion of Spinach molecules within a unit cell facilitates the pull-down of the RNAs by the capture oligonucleotide. In one single chip experiment, this method allowed us to create an array of 160 Spinach mutants with four repeats, including WT and negative controls. The coefficient of variation for the IVT of the Spinach mutants on the chip was less than 5% (Supplementary Figure S2), whereas the correlation coefficient between two experimental repeats was higher than 0.93 (Supplementary Figure S3).

For measuring the binding curves of DFHBI to the Spinach mutants, 13 concentrations of DFHBI were introduced to the chip in ascending order. Imaging of all unit cells occurred between the titration steps. Figure 1C shows an example of a fluorescence image from two neighboring unit cells with SPMs of Spinach G26A and A27G at the highest DFHBI concentration (43 μM). Figure 1D shows the typical resulting binding curve for the Spinach mutant A27G from a titration experiment. All Spinach fluorescence data that were obtained from the pull-down areas were normalized to the total amount of RNA by means of a probe molecule targeting the aptamer, which was introduced after the last DFHBI titration step.

The fluorescence curves of the titration experiment were fitted to standard mass law equations in order to obtain the dissociation constant, *K*_d_, of DFHBI toward the Spinach mutants and thus the free energy of binding, Δ*G*. After repeating the titration experiment at three temperatures, the binding enthalpy, Δ*H*, was calculated for each Spinach mutant by means of a Van't Hoff plot. Examples for the Van't Hoff plot are shown in Supplementary Figure S4. For this purpose the microfluidic chip was placed on a 0.1 mm thick glass slide coated with indium tin oxide (ITO). An applied electric current set the surface temperature of ITO glass slide (±0.25°C). The surface temperature of the chip was measured close to the surface with help of a temperature sensor introduced through a punch hole of the mLSI chip. Representative curves of Spinach mutant A27G's binding to DFHBI at 18°C, 23°C and 28°C are shown in Figure 1D. Furthermore, we tested whether the miniaturized RNA-small-molecule pull-down assay discriminated between the on and off chip binding data. The binding constant of WT Spinach toward DFHBI as assessed by the miniaturized pull-down assay, *K*_d_ = 1.18 μM, was slightly higher than the binding constant obtained for WT Spinach in a solution with and without the anchor sequence (*K*_d_ = 0.68 and 0.53 μM, respectively). The enthalpy of the WT Spinach/DFHBI binding was exothermic: 9 kcal/mol.

**Figure 2.**
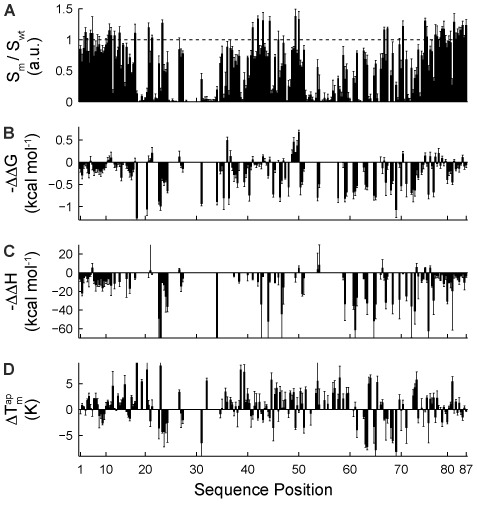
Single-point mutation (SPM) landscapes. (**A**) The normalized fluorescent signal of the Spinach mutant (*S_m_*) to the Spinach wt (*S*_wt_), (**B**) free energy (ΔΔ*G*), (**C**) enthalpy (ΔΔ*H*), and (**D**) Δ $T_m^{ap}$. of SPMs, where all values are relative to the WT Spinach. For each sequence positions of Spinach the two transversion and one transition mutation were included.

Figure 2A shows the fluorescence intensity of all SPM of Spinach at 23°C and at a DFHBI concentration of 43 μM. 76 SPM of Spinach showed no fluorogenic properties. 34 SPMs improved the fluorescent signal of Spinach, and a maximal increase of fluorescence by 1.3-fold was observed for the A75U mutation. In Figure 2B and C, we plotted the free energy and enthalpy landscapes of Spinach. Fluorescence intensity values and thermodynamic parameters for all Spinach mutants are given in the supporting information. The thermodynamic parameters are expressed as differences with WT Spinach. The highest increase of the Spinach/DFHBI binding affinity was observed around the sequence stretch of nucleotide 50, where the U50C mutation resulted in *K*_d_ of 0.45 μM, which translated into a decrease of ΔΔ*G* by 0.67 kcal/mol. Only for a few SPMs a minor increase in the binding enthalpy (∼5 kcal/mol) was observed within the enthalpy landscape.

Next to the thermodynamic landscape, the stability landscape of the DFHBI/Spinach complex can be determined using the same chip technology. To this end, we measured the apparent melting temperature of the RNA/DFHBI complex, $T_m^{ap}$, for the Spinach SPMs. Within the experimental series, the DFHBI concentration was held constant (43.5 μM), and the temperature was increased from 23°C to 49°C in 2°C increments. Examples of apparent unfolding curves of Spinach with SPMs are shown in Supplementary Figure S5. The stability landscape of Spinach (Figure 2D) is gained from the $T_m^{ap}$ values of all SPM of Spinach. According to the plot, approximately half of the mutations increased the $T_m^{ap}$ of Spinach. The largest increase of the $T_m^{ap}$ value of Spinach was 8.4°C measured for the SPM A23G. All data sets for Figure 2 are given in Supplementary Table S2.

**Figure 3.**
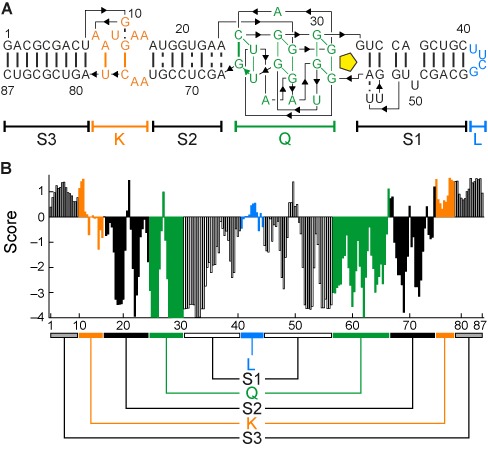
Landscape scoring for structural analysis. (**A**) The Spinach RNA structural elements based on the crystal structure, includes three stems (S1–S3), a knot (K), quadruplex (Q), and loop (L). (**B**) The scores sum up the landscape information on each SPM. The scores for base-pairing residues are similar, which explains the symmetry in the score plot.

Without *a priori* structural information on Spinach, the functional, thermodynamic, and stability landscapes of Spinach can be used to forecast structural RNA elements. To pinpoint key residues in the RNA structures, DPMs were included in the analysis. We wanted to investigate the possibility that a DPM can compensate for the effect of an SPM on base pairing in stem elements. Compared to the relatively low number of SPMs (261), the complete DPM scan required 11223 mutants for the 87-nt Spinach RNA. To simplify the structural screening of DPMs, the information on the four SPM landscapes was combined. For this purpose, each value within a landscape was scored against the maximum and minimum observed value (Eq. 7 and Supplementary Table S3). The sum of scored values (Eq. 8) was then filtered by a moving average function over the nearest SPM neighbors in 5′ and 3′ direction (Eq. 9) to obtain its local contribution to the fluorescence of Spinach. The result is shown in Figure 3B.

**Figure 4.**
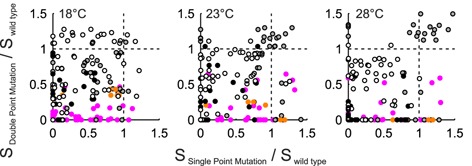
Functional screening of DPMs in Spinach. Perturbation and rescue of the Spinach fluorescence by a SPM and a DPM at three temperatures. Filled grey, white, black, orange and pink dots denote mutations in S3, S1, S2, K and control mismatched DPMs of Spinach, respectively.

Residues with positive or negative scores have beneficial or disadvantageous influence, respectively, on the overall integrity of Spinach. Clear symmetries were found within the score landscape of Spinach. For pairing of RNA bases in stem elements without direct binding to DFHBI, the same functional, thermodynamic, and stability profile of the overall structure was expected. Thus, sequence stretches with a similar score profile were highly likely to be related to each other. To prove this structural relationship, only tens of DPMs were now necessary rather than thousands. The compensatory effect of 91 DPMs on the RNA fluorescence at three temperatures is shown in Figure 4. DPMs based on the predicted 2D structure of Spinach were used as a control set. Here, 11 out of the predicted 25 DPMs were not confirmed by the crystal structure.

The plot of the SPMs against DPMs for the Spinach fluorescence demonstrated not only the compensatory effect but also that DPMs clustered within the structural elements of Spinach. Notably, the compensatory effects on the DFHBI binding and Spinach stability were more pronounced at higher temperatures (Supplementary Figure S6). This is because DPMs were tested only for structural rather than sequence influence, which was monitored with better sensitivity at temperatures close to the melting point of the unfolding transition. Beneficial thermodynamic traits of a DPM were utilized in the engineering section below.

According to the crystal structure of Spinach, we highlighted all nucleotides in stem elements within the score landscape (Figure 3B). The score profile of stems was reflecting known thermodynamic stability of stems, where a mutation had a smaller effect in the terminal regions than in the middle region of the stem. SPM mutations in S3 region do not negatively affect the Spinach fluorescence properties. This was previously observed and used to generate *Baby Spinach*, which is the minimal RNA sequence, by omitting the S3 stem (5). The G-quadruplex structure of Spinach was already visible within the fluorescence intensity landscape but was more pronounced in the score landscape with the lowest values for the G nucleotides around residues 30 and 60.

### Engineering analysis

**Figure 5.**
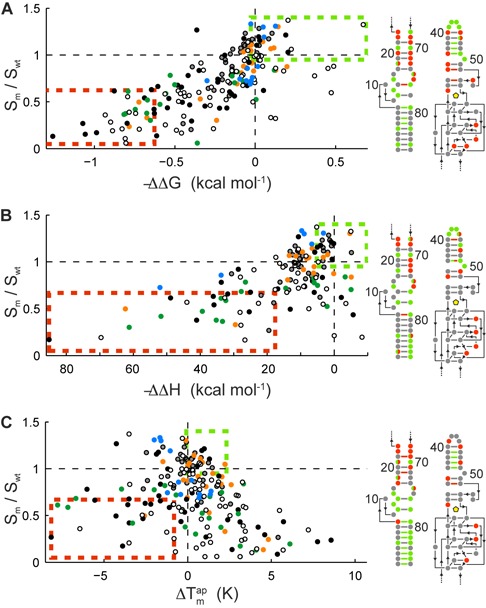
Identification of beneficial traits for engineering of Spinach variants. Correlation of the Spinach fluorescent signals with the (**A**) free energy of binding (ΔΔ*G*), (**B**) binding enthalpy (ΔΔ*H*), and (**C**) apparent melting temperature of the Spinach mutants in complex with DFHBI. All values are relative to WT Spinach. The color-coding in all panels corresponds to the structural elements of Spinach shown in Figure 3A. Mutations within the upper and lower 10% quantile are highlighted within a green and orange box, respectively. These beneficial and disadvantageous mutations are mapped on the 2D structure plot on the right with the same color code.

Fluorescence, thermodynamic binding, and stability landscapes are not only important for structural information on fluorogenic RNAs but are also used as an entry point for engineering the functional properties of fluorogenic RNAs. All mutants showing positive traits can improve fluorescent properties and modulate the binding affinity for the fluorogen. For selection of beneficial molecular traits, a multidimensional space correlation between the landscapes was utilized. Figure 5A shows the 2D correlation of the fluorescence intensity with the free energy of binding. With decreasing apparent binding affinity, the signal intensity of Spinach increases by 0.67 per kcal/mol. In the right-hand panel of Figure 5A, the 10% most beneficial mutants (leading to higher binding affinity) were matched to the Spinach structure. Just as with the free energy, a plot for the free enthalpy of binding (Figure 5B) and the fluorescent signal of Spinach for all mutations showed a negative linear correlation with a fold increase in fluorescence by 0.01 per kcal/mol. Although only a few mutations that we tested improved the binding enthalpy of Spinach, mapping SPM of the upper 10% quantile on the Spinach structure highlights the important enthalpy regions of Spinach. Finally, the correlation of $T_m^{ap}$ with the fluorescence intensity in Figure 5C shows a bell-shaped distribution rather than a linear correlation. In contrast to the binding parameters, the majority of mutations within the 10% quantile were DPMs. Beneficial SPMs (increasing the signal/binding relationship) were found in stem S2 and knob K, whereas stems S1 and S3 contained beneficial DPMs.

SPMs at the junction between the duplex and quadruplex structure of Spinach leading to a correction of the non-canonical base pair resulted in ∼20% enhancement of the fluorescent signal but little or no improvement in free energy of the DFHBI binding (±10 cal/mol). Nonetheless, such mutations had a strong negative influence on the binding enthalpy and thermodynamic stability (worse than −1.5°C) of Spinach. Various reports have shown that mismatched base-pairing at the DNA duplex-quadruplex junction determines the orientation of the junction between these two structural elements (23). The stability data here suggest that these two non-canonical base pairs are equally important for the duplex strand opening or orientation toward the G-quadruplex. A different picture is observed for the base mismatches upstream within the S2 stem (residues 18, 21 and 70). Here, SPMs leading to Watson–Crick base pairing improve the stability of the overall Spinach structure.

The tetraloop sequence UUCG with the C-G closing base pair of the WT Spinach is a frequently detected tetraloop in naturally occurring RNAs (24). At each tetraloop nucleotide position, one SPM led to a measurable increased of the Spinach fluorescence, where the largest signal was observed for the U42C mutation. The four beneficial SPM are either a pyrimidine/pyrimidine transition or purine/pyrimidine transversion and are given in Supplementary Figure S7A. Combination of two or four beneficial mutation at the tetraloop, however, led to clearly lower Spinach fluorescence signal (data not shown). Obvious was that a higher fluorescence signal led to a lower temperature stability of the DFHBI/Spinach complex. A comparison of the 12 tetraloops with published thermodynamic stability values is not possible due to missing corresponding experimental data. SPM leading to a mismatch in the closing and nearest neighboring pair of the tetraloop had strong negative effect on the Spinach fluorescence and temperature stability of the DFHBI/Spinach complex. All possible DPM in the closing and non-nearest neighbor base pair (Supplementary Figure S7B) showed higher temperature stability than the WT. Furthermore, the temperature stability of the Spinach/DFHBI complex followed widely published thermodynamic stability data for the closing and nearest neighbor base pair (24,25). In analogy to the SPM in the tetraloop DPM with stronger temperature stability of the Spinach/DFHBI complex correlated with a lower fluorescence signal, which argues that the binding pocket at the other end of stem1 requires a certain degree conformational freedom.

Measuring the thermodynamic stability of structural RNA elements in regions distant from the DFHBI binding sequence is only possible to a certain extent. This is exemplified through the exchange of the UUCG with evolutionally more prevalent GAAA and GUGA tetraloop sequence. For the GAAA we observed comparable stability and binding parameters of Spinach to DFHBI as for the UUCG loop. Introduction of the GUGA loop, however, led to the disruption of the Spinach/DFHBI complex due to misfolding, which was indicated by loss of the fluorescence at lower temperature.

Thermodynamic base pair fingerprints of the binding center itself cannot be obtained due to the immediate loss of the fluorescent signal during the DFHBI binding, except for residues 27, 59, 61 and 63. According to the crystal structure, residue 61 functions as a ‘gateway’ for DFHBI and the three residues 27, 59 and 63 protrude from the binding site. Mutation of the uracil and adenine residues to cytosine or guanine at these positions reduces the binding affinity and apparent folding temperature of the RNA/DFHBI complex strongly. One possible reason for this could be the formation of concurrent base pairing reactions with residues of the G-quadruplex structure within the mutants.

**Figure 6.**
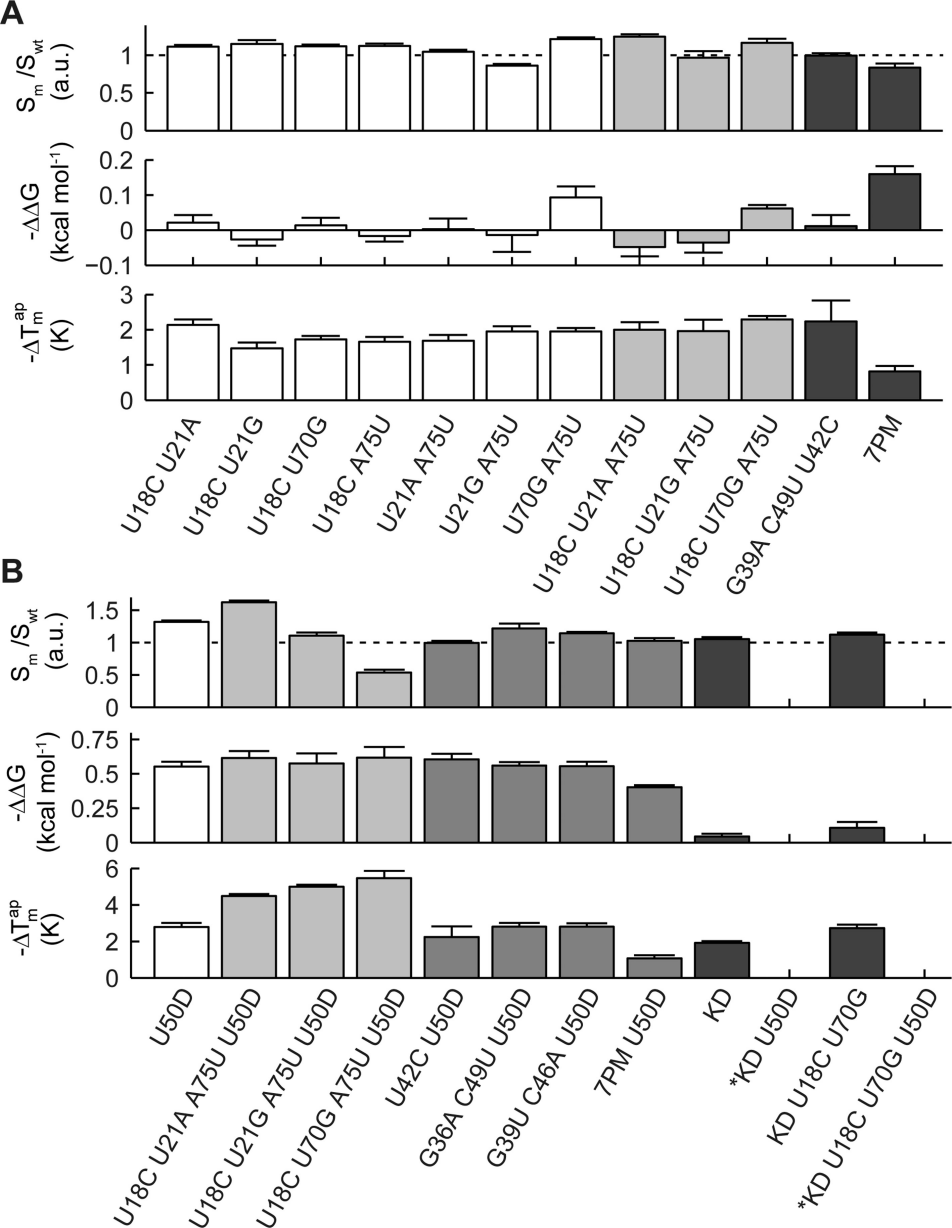
Thermodynamics profiles of combined beneficial spinach point mutations. (**A**) Combinatorial effect of two, three and seven beneficial point mutations on the functional and thermodynamic profile of Spinach. The 7 point mutation (PM) includes the mutations A12G, A15G, U18C, U21A, G36A, C49U, A75U. (**B**) Combination of the beneficial point mutations with the deletion of the U50 nucleotide (U50D) and knob region (KD). Combinatorial mutations of Spinach marked with an asterix showed a bimodal melting curve.

To demonstrate that our analysis can be used to improve the Spinach function and stability, we selected the SPM mutations, which present in all top 10% quantile of the binding, temperature and fluorescence parameters. The selected SPM mutants were then combined by synthesizing Spinach sequences with double, triple and seven point mutations. The thermodynamic results are shown in Figure 6A. Beneficial traits of the single mutants are not additive but improve the thermodynamic profile stronger than the single mutant alone. Additionally, we combined beneficial point mutations with the deletion of the U50 residue and the knob structure element. The deletion of the U50 base has been suggested previously for enhancing Spinach stability, which is supported by our finding above. The ΔΔ*G and* Δ $T_m^{ap}$ for the engineered Spinach RNA molecules are given in Figure 6B. Deletion of the knob and thus merging of S2 and S3 has only small benefit for the stability of Spinach. Deletion of the U50 nucleotide improved the binding affinity of Spinach to DFHBI in the same order as the U50C mutant. In difference to the base mutation, the base deletion increased the melting temperature of the Spinach/DFHBI complex strongly by 2.8°C. Together with the accumulative point mutations, the U50 deletion could raise the Spinach fluorescence signal by a factor of 1.6, the Δ*G* of Spinach to DFHBI by 0.61 kcal/mol, and the $T_m^{ap}$ by 4.5°C. The $T_m^{ap}$ is greater than the improvement from the first generation of Spinach to the second (Spinach-2), which mainly involved point mutations in S3. It is important to note that not all accumulative point mutations led to an increase. A negative example was the combination of the deletion of the knob region and the U50 nucleotide, which destroyed the fluorescence properties of Spinach at 25°C. This effect can be explained by a folding problem of the Spinach mutant. This is confounded on the finding of a bimodal melting curve for this mutant, where no fluorescence was detected at low and high temperatures and a moderate fluorescence around 35°C (see Supplementary Figure S7 C). The fluorescence of the published Baby Spinach sequence, in which also the knob region and U50 nucleotide was deleted retaining the fluorescence properties of Spinach at 25°C (5). A direct comparison, however between the two mutants is not possible since the KD U50D Spinach mutant here still contains the S3 stem. With these observations, it becomes clear that an iterative testing is required for improving aptamer properties based on SPM results. In contrast to the evolutional optimization step from Spinach 1 to Spinach 2, our directed approach also increased the fluorescence intensity and binding affinity of Spinach to its native fluorogenic compound. Point-to-point comparison of the mutations between Spinach2 and our version is given in Supplementary Figure S8.

## CONCLUSION

With the complete thermodynamic binding and stability landscapes of the truncated version of the first-generation Spinach–DFHBI complexes available, we demonstrated that it is possible to optimize longer RNA molecules by the mLSI chip technology. The current development of next generation fluorogenic ligands and RNA sensors by various evolutional approaches (1–3) can be assisted and guided by the corresponding full thermodynamic profiles. Direct engineering of RNA functionality is expected to be most effective in a later process state or the assembly of multiple RNA elements into RNA switches (9,12,14,26) or RNA circuits (10,11). For example, the free-energy binding landscape of our Spinach revealed mutants with affinities for DFHBI spanning two orders of magnitude. Fine-tuning of ligand affinity of fluorogenic sensors is a central task during the adaptation of RNA sensors to different physiological working ranges. Although the *in vitro*-designed sequences may not live to expectations *in vivo*, they are fully applicable to analytical sensor systems on microfluidic chip platforms. The complete thermodynamic binding and stability landscape serves as a guide for decisions to add new functional RNA elements to a minimal fluorogenic Spinach sequence for optimization of their combined functions. Furthermore, the fluorescence sensitivity of Spinach itself in conjunction with the microfluidic chip can be used to systematically screen Spinach variants for stability of distal bridges or of switch sequences fused at the end of either the S1 or S2 stem. In perspective the presented microfluidic approach is a promising method for rational design of fluorogenic RNAs.

## Supplementary Material

SUPPLEMENTARY DATA
