# Supplementary material for: Systematic reconstruction of binding and stability landscapes of the fluorogenic aptamer spinach
Source: Nucleic Acids Res. 2015 Sep 22;43(19):9564–72. doi: 10.1093/nar/gkv944 (PMC4627091; doi:10.1093/nar/gkv944)
Supplement: SUPPLEMENTARY DATA [file supp_43_19_9564__index.html]

Systematic reconstruction of binding and stability landscapes of the fluorogenic aptamer spinach — SUPPLEMENTARY DATA 

# Systematic reconstruction of binding and stability landscapes of the fluorogenic aptamer spinach

## SUPPLEMENTARY DATA

- SUPPLEMENTARY DATA
- SUPPLEMENTARY DATA
