# Supplementary material for: Systematic reconstruction of binding and stability landscapes of the fluorogenic aptamer spinach
Source: Nucleic Acids Res. 2015 Sep 22;43(19):9564–72. doi: 10.1093/nar/gkv944 (PMC4627091; doi:10.1093/nar/gkv944)
Supplement: SUPPLEMENTARY DATA [file supp_gkv944_nar-01673-r-2015-File008.pdf]

Simon Ketterer[1,2], David Fuchs[1,2], Wilfried Weber[2,3], and Matthias Meier[1,2,*]

Systematic Reconstruction of Binding and Stability Landscapes of the Fluorogenic Aptamer Spinach

SUPPLEMENTARY INFORMATION

Supplementary Tables

Table S1. Oligonucleotide sequences

TF	DNA Binding Sequence
WT O1	5'-GCTAATACGACTCACTATAGGGAACACAATAATAAAACTTACAAACAAGAAAA AAAAGACGCGACTGAATGAAATGGTGAAGGACGGGTCC-3'
WT O2	5'-GACGCGACTAGTTACGGAGCTCACACTCTACTCAACAAGCTGCCGAAGCAG CTGGACCCGTCCTTCACCATTTCATTCA-3'
FP	5'-GCTAATACGACTCACTATAGGGAACACAAT-3'
RP	5'-GACGCGACTAGTTACGGAGCTC-3'
Control RP	5'-CTTGTTTGTAAGTTTTATTATTGTGTTCC-3'
Anchor	[ROX]-5'-CTTGTTTGTAAGTTTTATTATTGTGTTCC-3'-[BtnTg]
Probe	5'-GTCCTTCACCATTTCATT-3'-[Cy5]

Table S3. Minimal and maximum values used for normalization of the thermodynamic and brightness data in the scoring plot (Figure 3).

	S a.u.	$-\Delta G$ kcal/mol	ΔH kcal/mol	T_m^{ap} K
x_max	0	-1.26	-85.14	-8.15
x_min	1.37	0.67	8.30	10.69

Table S4. Comparison of the thermodynamic data of the different Spinach generations.

	K_D	T_m^{ap}	Relative Brightness	Relative Signal Intensity
Spinach[1]	450 ± 12	34 ± 0.6	1	NA
Spinach wt (used in here)	678 ± 2	38.5 ± 0.6	NA	1
Spinach 2[2]	430 ± 12	38 ± 0.4	1.03	NA
Baby-Spinach[3]	NA	NA	0.95	NA
Improved Spinach	240 ± 2	43.0 ± 0.2	NA	1.6

REFERENCES

1. Paige, J.S., Wu, K.Y. and Jaffrey, S.R. (2011) RNA mimics of green fluorescent protein, *Science (New York, N.Y.),* **333,** 642–646.
2. Strack, R.L., Disney, M.D. and Jaffrey, S.R. (2013) A superfolding Spinach2 reveals the dynamic nature of trinucleotide repeat-containing RNA, *Nature methods,* **10,** 1219–1224.
3. Warner, K.D., Chen, M.C., Song, W., Strack, R.L., Thorn, A., Jaffrey, S.R. and Ferré-D'Amaré, A.R. (2014) Structural basis for activity of highly efficient RNA mimics of green fluorescent protein, *Nature structural & molecular biology,* **21,** 658–663.

Supplementary Figures

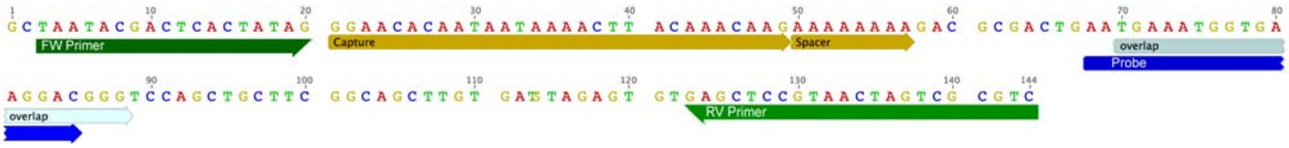

Figure S1. DNA sequence for on chip Spinach transcription and interrogation. Colored arrows below the sequence indicate the functional elements for capture and detection of the RNA aptamer (see also Table S1).

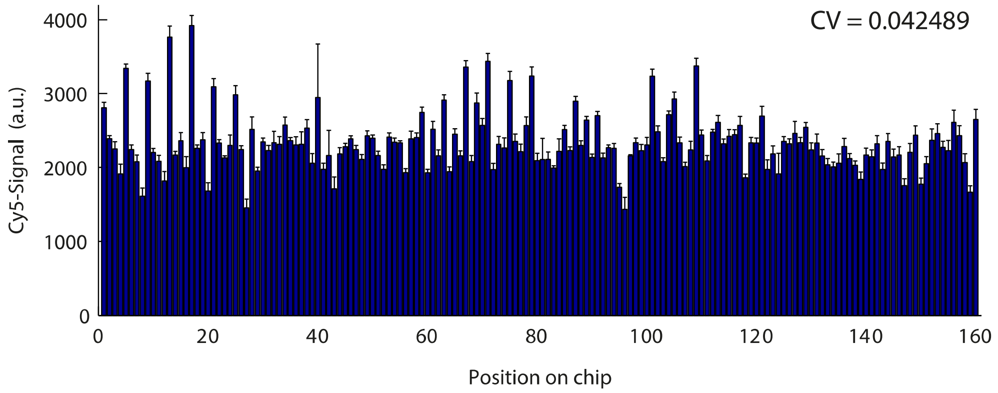

Figure S2. *In vitro* transcription analysis of 160 Spinach mutants on chip. The plot shows the fluorescence probe signal after hybridization to the 160 *in vitro* translated and pull-down Spinach mutants. The coefficient of variation (CV) between the mutants was 0.042.

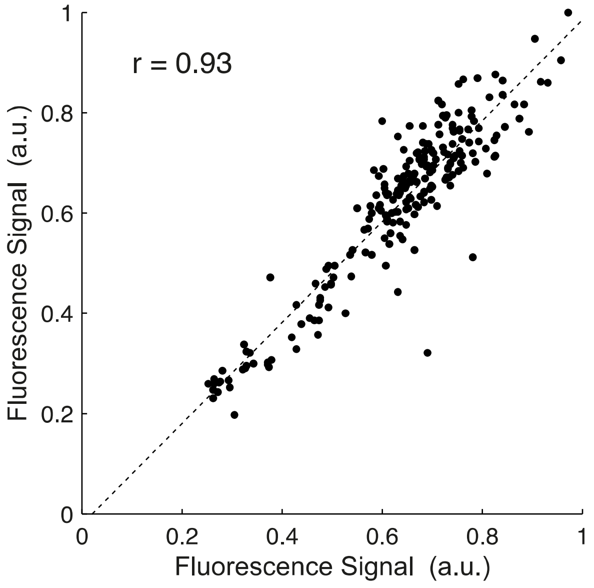

Figure S3. The fluorescence signal of 160 SPM of Spinach measured in two microfluidic chip experiments under the same conditions (43.5µM DFHBI, 23°C).

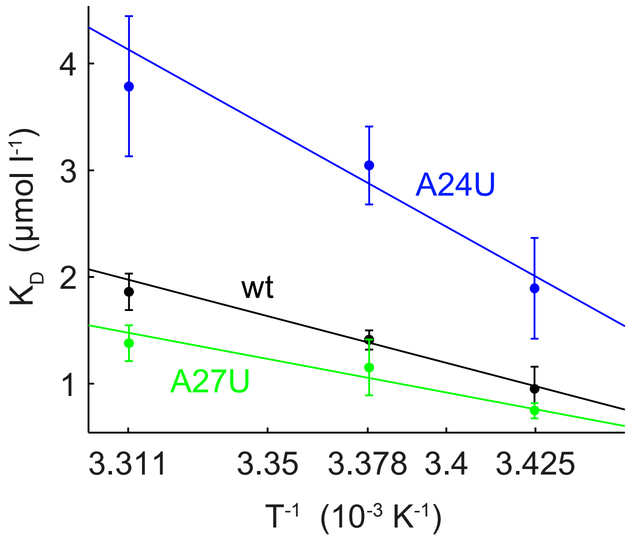

Figure S4. Representative van’t Hoff plot of *wt* Spinach and the SPM of A24U (blue) and A27U (green) obtained from the chip measurements.

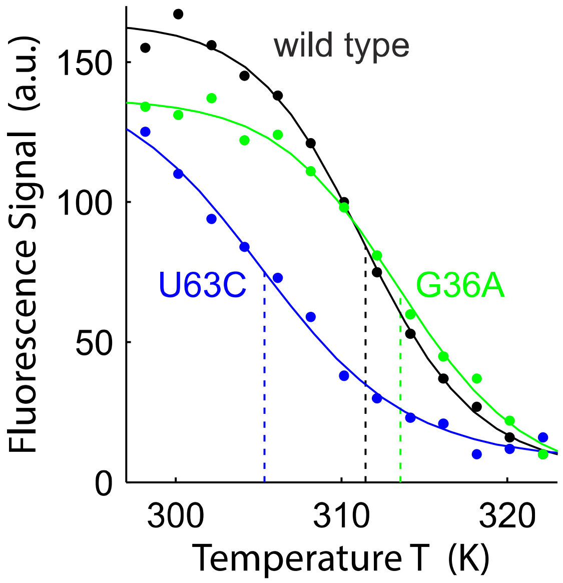

Figure S5. Representative melting curves of *wt* Spinach and the SPM of U63C (blue) and G35A (green) obtained from the chip measurements.

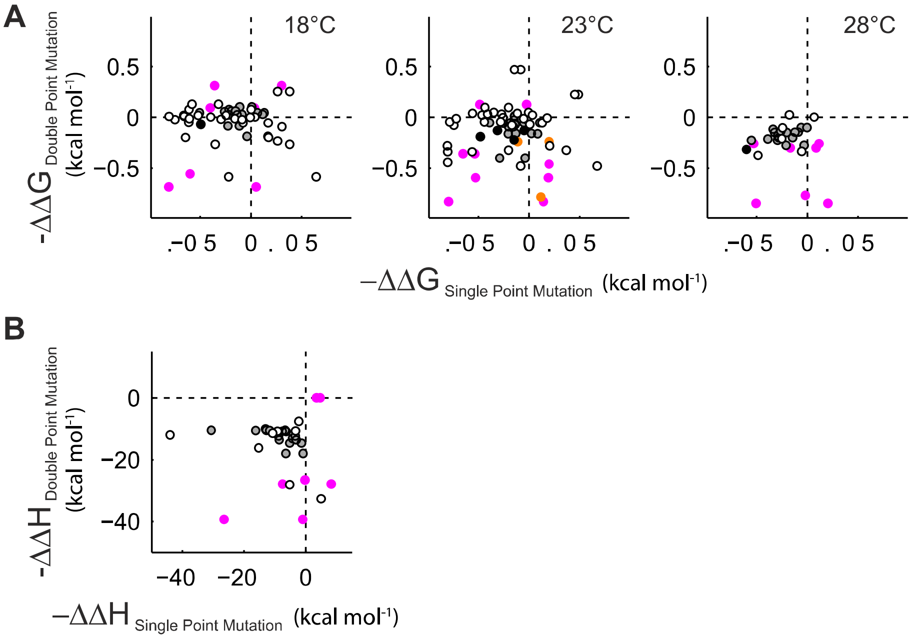

Figure S6. Thermodynamic screening of double-point mutations (DPMs) in Spinach. Perturbation and rescue of the Spinach ΔΔG and ΔΔH by a single-point mutation (SPM) and a DPM at three temperatures. Filled grey, open grey, black, orange, and pink dots denote mutations in S3, S1, S2, K, and control mismatched DPMs of Spinach, respectively.

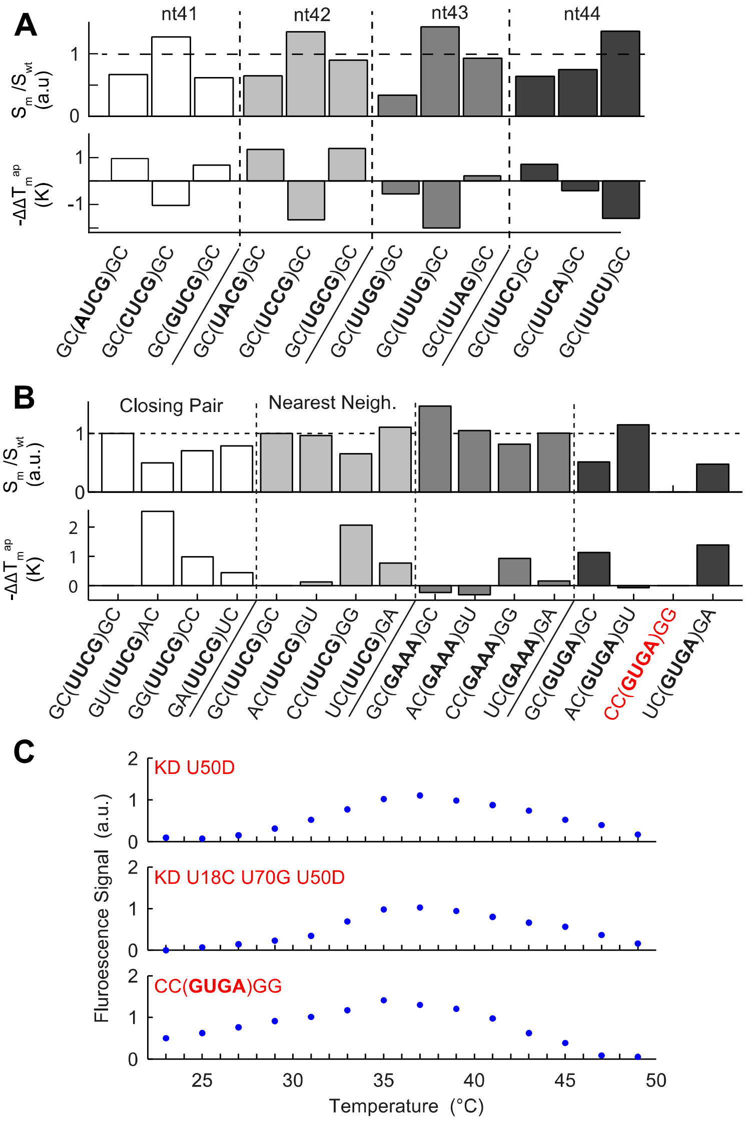

Figure S7. Temperature stability and fluorescence signal of the Spinach/DFHBI complex upon introducing single and double point mutations within the tetraloop. A) SPM within the nucleotide positions 41 to 44. B) DPM within the closing and nearest neighbor of nucleotide pair of stem1. Further, exchange of the UUCG to GAAA and GUGA tetraloop sequence. The mutant highlighted in red showed a bimodal melting curve. C) Bimodal melting curve of three Spinach mutants indication a folding problem of the Spinach structure.

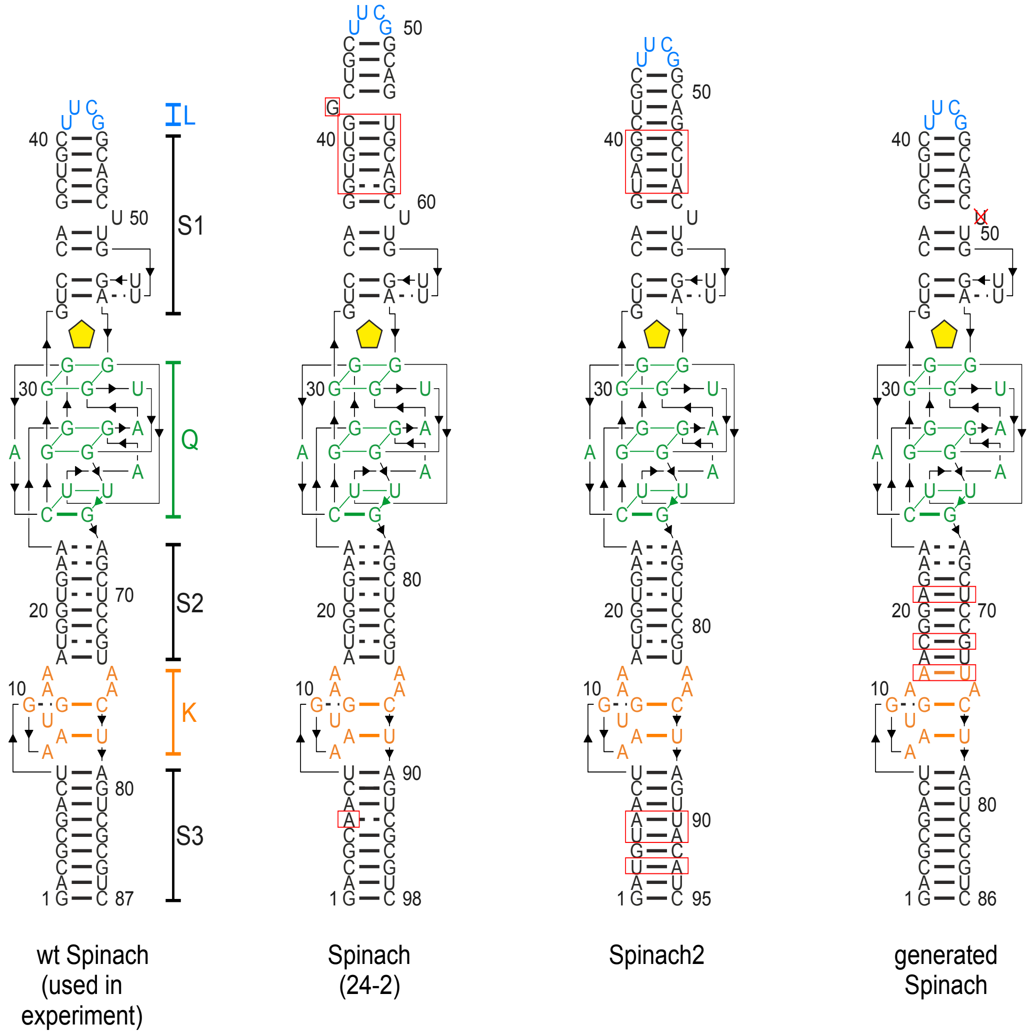

Figure S8. Sequence comparison of the different Spinach generations established by evolutional approaches (Spinach 24-2 and Spinach2) to the wt Spinach version used in here (left) and optimized Spinach generation (right) evolved by directed engineering within this manuscript.
